# The effect of proton irradiation on the properties of a graphene oxide paper†

**DOI:** 10.1039/c9ra05389a

**Published:** 2019-09-26

**Authors:** Xiaojuan Zhen, Yifan Huang, Shengsheng Yang, Zhanzu Feng, Dedong Ba, Jianhong Zhuang, Yi Wang, Xiaogang Qin

**Affiliations:** Lanzhou City University 730000 China; Lanzhou Institute of Physics 730000 China; Science and Technology on Vacuum Technology and Physics Laboratory 730000 China; National Key Laboratory of Materials Behavior and Evaluation Technology in Space Environment 730000 China

## Abstract

A graphene oxide paper (GOP) was irradiated with 500 keV proton for total fluence of 2 × 10^13^ cm^−2^ to 2 × 10^15^ cm^−2^ in a ground-based irradiation simulator. The spacing of layer, surface chemical composition, structural defects, thermal conductivity and electrical property of the GOP before and after irradiation was measured. The results indicated that the spacing of layer decreased after irradiation. The ratio of total carbon atom and total oxygen atom increased from 2.40 to 4.31 as well as the sp^2^ hybridized carbons obviously increased after 2 × 10^15^ cm^−2^ irradiation. The XPS analysis suggested the occurrence of reduction, and the Raman spectra indicated that the defects were produced after proton irradiation. Furthermore, the thermal conductivity of GOP decreased, and then increased smoothly as the irradiation fluences were increased, and the electrical property showed the similar trend. The change in the thermal and electrical properties for GOP could be attributed to the defects and the removal of oxygen-containing functional groups, which lead to the phonon conduct path and scattering centers changed under proton irradiation. This study could promote the application of GOP in future space expeditions.

## Abstract 2

The 500 keV proton irradiaiton effect on a graphene oxide paper was studied in a ground-based irradiation simulator.

## Graphical abstract

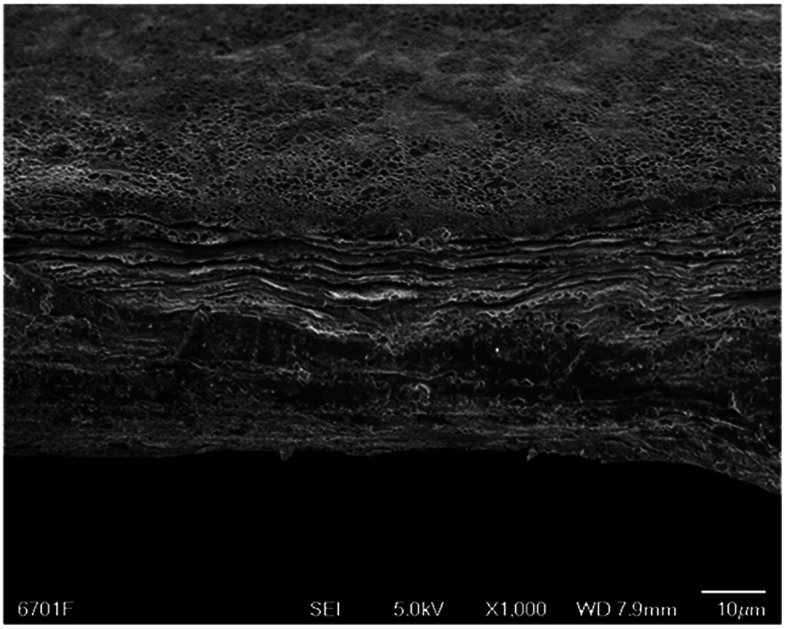

## 1. Introduction

The extraordinary physical and mechanical properties have raised concern about two dimensional graphene materials, which exhibit outstanding characteristics in the aspect of thermal conductivity, high carrier mobility and ballistic electron transport, good fracture and mechanical strength, chemical inertness and large specific surface area.^1^ This emerging nano carbon material has brought about extensive attention in many research fields, including stored energy, catalysts supports, field-effect transistors, super capacitors, memory cells, optoelectronic.^2^ Based on their many exceptional aspects, such as light weight, graphene is also getting attention due to its unique requirements in aerospace. NASA proposed that graphene can be used as nanocomposites, charge dissipation layers and multifunctional electronic components in spacecraft.^3^ Zhang *et al.* has demonstrated that graphene can be used as protection coatings under atomic oxygen erosion.^4,5^ The study of ultraviolet exposure effects on graphene and its field effect transistors are important keys on the design of electronic and optoelectronic devices for space missions. Emilie J. Siochi pointed out that the graphene used in aerospace mainly depends on reliable functionality like any other new material,^3^ so it is necessary to study the irradiation effect, such as gamma ray, energetic particles from space environment before they will be used in aeronautics and space applications.

The existing irradiation effect research on graphene is mainly focused on materials preparation and property modification, and the experiment materials used are small-sized or thin films. For instance, some studies show that the gamma ray irradiation is an effective and green approach for the reduction, functionalization, the synthesis of nanocomposites and the self-assembly of oxide graphene sheets.^6^ The majority of research for energetic particles, including ion and electron effects on graphene, includes modification in property and structure.^7–12^ Chen *et al.* found that graphene oxide (GO) can be partially reduced by electron beam irradiation,^12^ and the adsorptive performance of the reduced graphene oxide (rGO) also can be improved.^13^ The research indicates that ion irradiation is a useful way for the modification of GO and also for tailoring its structure,^14–16^ and the electron emission properties can be enhanced by irradiation.^14^ Tyagi *et al.* has exhibited in their published study that the maximum lattice temperature of a GO film (GOF) can be changed by C ion irradiation.^17^ The changes brought in structure and property was defects, orders of magnitude and amorphization induced by irradiation. These researches have an undeniable role in promoting the space application of graphene, though the initial intention was not based on this aim. There are also some studies based on the space radiation effect on graphene materials, in which the materials in the experiments are usually suspended graphene, with size as the limitation.^11,18^

The charged particles from near-Earth space and solar cosmic rays, including high-speed protons, are a threat to astronauts and satellites. The most damaging effects are known to be the charging of the surfaces and electrostatic discharge in the spacecraft. The study of proton irradiation damage in a mono-layer graphene suggests that the energy/dose has a direct relationship on defect formation.^18^ The research by Chen *et al.* shows that the structural defects and electrical characteristics of graphene transistors change after different energy proton irradiations.^19^ Mathew *et al.* have also found that the stability and the layer number of graphene on a SiO_2_/Si substrate fabricated by micro-mechanical exfoliation are influenced by focused proton irradiation.^20^ Therefore, there is lack of irradiation effect on the bulk graphene materials synthesized by a chemical method, which is considered to be a promising method for mass production. For this, the proton effect on bulk GO materials has been studied in this paper.

A free standing GO paper (GOP) is a fascinating material possessing excellent mechanical properties and chemical stability, and its potential applications cover isotropic ionic conductors, thermal conductive films, mechanically reinforced composites or transparent materials.^9,10,15,21^ In the AP-8_max_ trapped proton environment of the Earth's radiation belt, the proton irradiation energy is in the range of 0.1–400 MeV.^22^ The current research focussed on the 5–20 MeV proton irradiation on a monolayer or a thin film graphene,^18,23,24^ and Korkut *et al.* have studied the 500 keV proton irradiation on supported bilayer graphene by a simulation method.^25^ In this study, we have adopted the AP-8_max_ mode to discuss the 500 keV proton effect irradiation on bulk GOP. The total purpose is to promote the bulk graphene in harsh space applications.

## 2. Experimental

### 2.1 GOP preparation

GOP was purchased from JCNANO company in China with the thickness of about 50 ± 10 μm, size 6 cm × 6 cm and the density of 1.30 g cm^−3^, respectively, which was obtained by the Hummers' method. The materials were vacuumed for 24 h, which were maintained at 0.1 Pa to prevent contamination and ensure that the desorption processes finished.

### 2.2 Proton irradiation experiment

The proton irradiation experiment was performed using a ground-based irradiation simulator at Lanzhou Institute of Physics (LIP), China Academy of Space Technology (CAST) 730000. (CAST, Lanzhou, China). The irradiation of the samples was conducted by 500 keV proton with the flux of 1 × 10^10^ cm^−2^ s^−1^ for ion fluences 2 × 10^13^, 2 × 10^14^ and 2 × 10^15^ cm^−2^. The pressure was kept in vacuum of 10^−5^ Pa at room temperature in an irradiation chamber.

### 2.3 Material characterization techniques

To study the proton irradiation on GOP, the X-ray diffraction (XRD) were conducted using Cu Kα radiation (*λ* = 0.154 nm) with a rate of 5° min^−1^ at room temperature. The X-ray photoelectron spectroscopy (XPS, EscaLab 250 Xi spectrometer) was performed to determine the chemical state of the GOP. The laser Raman spectroscopy was performed using an HR Raman spectrometer 532 nm laser with Nd filter 0.1% to get the information about the structure composition. The thermal diffusion coefficient of GOP was measured using a thermal conductivity meter (LFA laser flash apparatus, Netzsch LFA 467) in plane. The fracture surface of a pristine GOP was observed using a field emission scanning electron microscope (FE-SEM, Hitachi S4800). The electrical property of GOP was investigated by square resistance test with a four-point probe (RTS-9 KEITHLEY 2400). The XRD and XPS experiments were performed at Lanzhou university, and the Raman spectroscopy and SEM tests were performed at Lanzhou Institute of Chemical Physics, Chinese Academy of Sciences, and the thermal conductivity test were performed at NETZSCH Scientific Instruments Trading (Shanghai, China), respectively. The test results of GOP before and after proton irradiation were evaluated and compared.

## 3. Results and discussion

### 3.1 X-ray diffraction (XRD) analysis

**Fig. 1:**
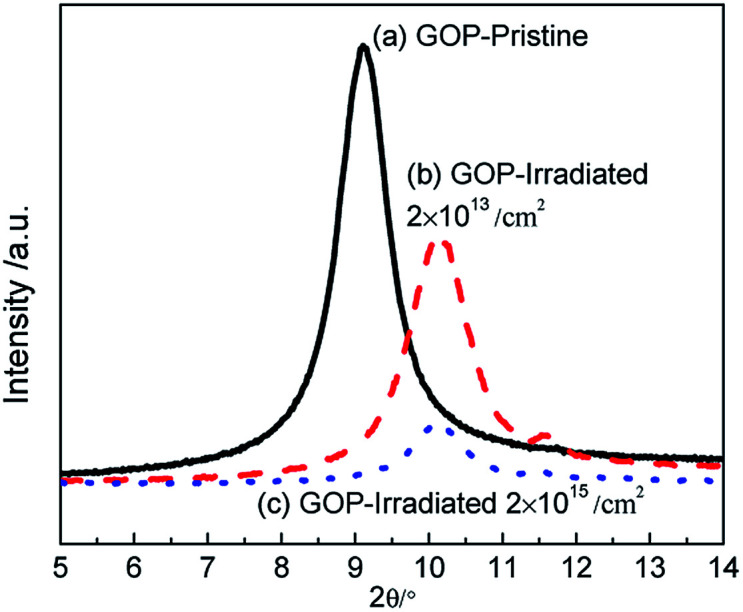
The XRD patterns of pristine and irradiated GOPs.

The XRD patterns of GOP before and after irradiation are shown in the inset of Fig. 1. It can be seen that the XRD patterns of the pristine GOP display a sharp and intense peak located at 9.10°, and the corresponding *d*-spacing (the distance of layer-to-layer) is 0.48 nm according to the Scherrer's equation as show in expression (1). The diffraction peak gradually shifts to 10.14° with the *d*-spacing 0.44 nm after proton irradiation with the fluences 2.0 × 10^13^ and 2.0 × 10^15^ cm^−2^. The intensity of the domain peak decreased dramatically, which may be due to the removal of oxygen-containing functional groups accompanied with the edges and basal plane of materials, and the increase in broadness of diffraction peaks with irradiation fluences indicates a disordered structure.^7,12,26,27^1
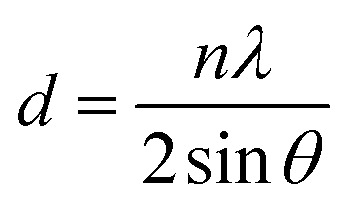
where *d* is the layer-to-layer distance, *n* is the order, *λ* is the wavelength of X-ray and *θ* is the corresponding angle of XRD peak.

### 3.2 X-ray photoelectron spectroscopy (XPS) analysis

**Fig. 2:**
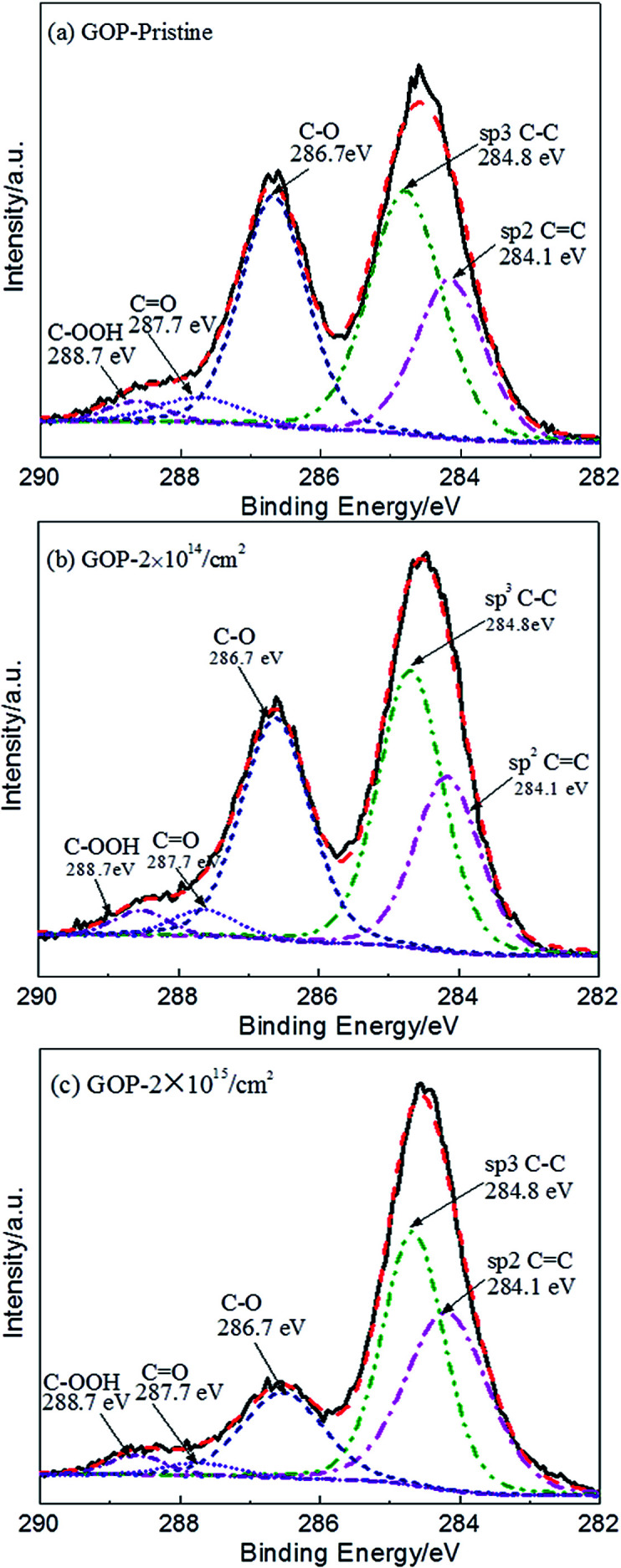
XPS results of the pristine GOP (a) and the irradiated GOP with the fluences of (b) 2 × 10^14^ cm^−2^ and (c) 2 × 10^15^ cm^−2^ under the 500 keV proton irradiation, respectively.

To further study the proton effect on GOP, the changes in the functional compositions are analyzed by XPS, and the C 1s peaks are divided into different curves according to symmetric Gaussian. Fig. 2 is the C 1s peaks of the pristine and GOP after irradiation, respectively, and the results are presented in Table 1. The GOP before and after irradiation show five kinds of binding energies in Fig. 2(a)–(c). The dominant peak at 284.1 eV is attributed to the existence of sp^2^ hybridized carbon (C=C bond) as well as sp^3^ hybridized carbons (C–C, 284.8 eV), and other peaks, including C–O bond (hydroxyl and epoxy), C=O bond (carbonyl) and C–OOH bond (carboxyl) appears around 286.7 eV, 287.7 eV and 288.7 eV, respectively. A similar result has been reported for others.^10^ It can be seen from Fig. 2(b) and Table 1 that the 2 × 10^14^ cm^−2^ irradiated GOP show a smooth change in functional compositions, while the total carbon to total oxygen (C/O) atomic ratio increased to 2.77. When the irradiation fluences increase to 2 × 10^15^ cm^−2^, the content of oxygen-containing functional groups decreased from 39.58% for the pristine GOP to 23.06% for the irradiated GOP, while value of C/O atomic ratio was increased from 2.30 to 4.31. Moreover, the ratio of the sp^3^ hybridized atoms and the sp^2^ hybridized atoms was decreased by 34.35%. The C–O bond and the C=O content was decreased appreciably, whereas the COOH bond content gradually increased. The decrease in hydroxyl, epoxide and carbonyl indicates the reduction action that happened in GO,^6,28–31^ whereas we speculated that the GOP was highly reduced under higher irradiation fluences. Meanwhile, it is suggested that the C–O components was converted to sp^2^ hybridized carbon under energetic proton knocking on carbon atoms. Moreover, the sp^2^ hybridized carbon was easily formed than sp^3^ hybridized carbon under proton irradiation.

**Table 1:** The results of XPS analysis for pristine and irradiated GOP

Groups	Binding Energy (eV)	Pristine	2 × 10^14^ cm^−2^	2 × 10^15^ cm^−2^
		Content (%)		
sp^2^ C=C	284.1	22.96	23.68	37.15
sp^3^ C–C	284.8	37.46	37.55	39.79
C–O	286.7	32.35	32.27	17.76
C=O	287.7	4.66	3.63	2.40
C–OOH	288.7	2.57	2.87	2.90
sp^3^/sp^2^	—	1.63	1.59	1.07
C/O	—	2.30	2.77	4.31

### 3.3 Raman spectroscopy analysis

**Fig. 3:**
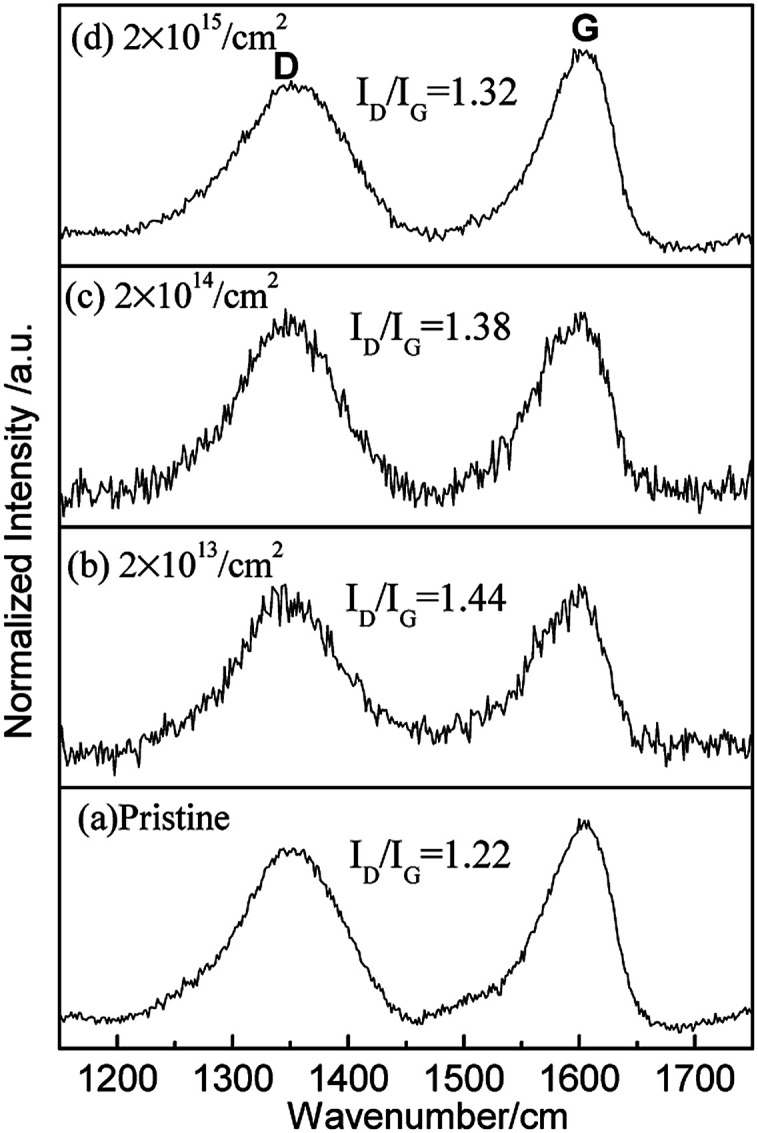
Raman spectra of the pristine and irradiated GOP.

Raman spectroscopy is used as a common tool for studying the structural composition of GOP, which is an invaluable method in graphene studies.^10,29,31–36^ It can be used to confirm the vibrational modes of surface functional groups and structural damage. Fig. 3 shows the Raman spectra of GOP for the pristine and irradiated samples. Both of the spectra show a D peak positioned at 1345 cm^−1^ and a G peak positioned at 1590 cm^−1^. The G band indicates the origin of the stretching vibration of the sp^2^ carbon atoms at the Brillouin zone center and its existence is considered neglecting the order or disorder of sp^2^ carbons in the material.^37–40^ In a perfect graphite structure, the vibrational mode associated with D peak is not permitted and only appears for six-fold carbon rings located near structural defects. Therefore, the observation of D peak is a common method for the detection of defect formation in carbon materials, and the D band is representative of defects, such as bond-angle disorder, bond-length disorder, vacancies, edge defects, amorphous, involving the transverse optical phonons near the *κ*-point phonons. Therefore, the ratio of the peak intensities *I*_D_/*I*_G_ (D peaks and G peaks) is a typical analysis method to evaluate defect formation and the structure order in a qualitative way. The strong D peak refers to the defects produced during fabrication and the influence of structure edges in the pristine GOP.^31^ For pristine GOP irradiated with different fluences, the value *I*_D_/*I*_G_ was first increased from 1.22 to 1.44 and then decreased to 1.32. Furthermore, the FWHM of the D and G peaks for pristine is 95 cm^−1^ and 66 cm^−1^ but then increases to 106 cm^−1^ and 70 cm^−1^ after the irradiation of 2 × 10^15^ cm^−2^ fluences, respectively. The results indicate that the large numbers of defects are created in the lower 2 × 10^13^ cm^−2^ and 2 × 10^14^ cm^−2^ fluences irradiated GOP. The D and G peaks are wide in higher 2 × 10^15^ cm^−2^ fluence irradiated GOP, and it can be concluded that the disorder structure is formed, and the higher value *I*_D_/*I*_G_ suggest the C=C bond (sp^2^ carbon) distortion, and the non-six ring carbon rings production.^18,29,31,35,38^

In general, the *I*_D_/*I*_G_ value is very sensitive to defects and six-ring structure,^26^ while the broadening of the FWHM of D and G peaks always represent a structure disorder.^41,42^ Many previous studies suggest that there is a positive correlation relation between the number of defects and *I*_D_/*I*_G_ ratio in graphene.^18,43^ In this study, the *I*_D_/*I*_G_ value increases obviously after 2 × 10^13^ cm^−2^ irradiation fluences in GOP, which indicates that the proton irradiation lead to a high defect density. In the 2 × 10^15^ cm^−2^ fluence irradiated GOP, the decline in the *I*_D_/*I*_G_ value suggests the lower defect density compared to lower fluence irradiated materials, which is attributed to the complete removal of oxygen-containing functional groups. Combined with the above XRD and XPS analysis, the findings indicate that although the amount of oxygen-containing functional groups is reduced compared to that in pristine GOP, many defects are retained after the high fluence irradiation process.^10,18,23,29,32–36,41,43^

### 3.4 Thermal conductivities

Recently the thermal properties of lower dimensional graphene have attracted considerable interest among scientific and engineering communities. The heat transport is a key factor for the graphene in the electronic industry. Therefore, the thermal performance of GOP has been studied in this study. The thermal diffusivity in plane of the sample can be directly obtained by LFT transient method. The circle shaped sample with the diameter of 2.5 mm is heated from one side, and then the temperature change is obtained on the opposite side. The thermal conductivity is obtained according to the eqn (2).

**Fig. 4:**
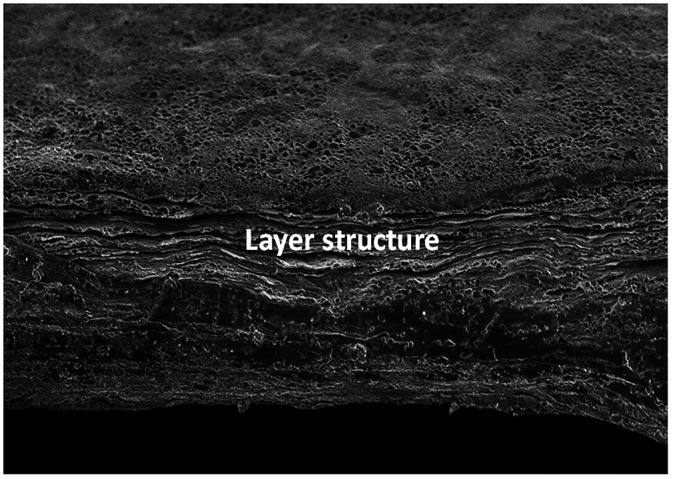
SEM images of pristine GOP from the cross-sectional view.

2 *k* = *αC*_p_*ρ*

where *k* W (m K)^−1^ is the thermal conductivity that needs to be calculated, *α* is the thermal diffusivity, *C*_p_ J (g K)^−1^ is the specific heat capacity assumed as value 0.708,^42^ and *ρ* (g cm^−3^) denotes the volume density of the samples. In order to get the *ρ*, weight of the GOP is measured in a microbalance, and the thickness is measured by SEM. The average value of the thickness of GOP is obtained from the cross-section structure that can be observed in Fig. 4. From the microstructure of the cross-section, it can be found that the materials have layered structure.

Table 2 shows the thermal diffusivity and thermal conductivity in plane. It can be seen that the thermal conductivities of pristine GOP is 2.795 W (m K)^−1^ , which may be caused by the lower quality of a layer structure and due to the influence of the defects. The thermal property of irradiated GOP shows that the thermal conductivity is decreased mildly and then increased to 2.982 W (m K)^−1^with the irradiation fluences of up to 2 × 10^15^ cm^−2^. The thermal transport in carbon materials is mainly determined by acoustic phonons, and the phonon can be further influenced by the defects and structure disorder.^44–49^ The defects, disorder structure and the oxygen functional group are influenced by proton irradiation, leading to the change in the thermal properties of GOP.

**Table 2:** The thermal conductivity results of GOP before and after proton irradiation

Sample	Irradiation fluences	Thermal diffusion	Thermal conductivity
Pristine	0	3.058 m^2^ s^−1^	2.795 W m^−1^ K^−1^
Irradiated	2 × 10^13^ cm^−2^	2.980 m^2^ s^−1^	2.723 W m^−1^ K^−1^
Irradiated	2 × 10^14^ cm^−2^	2.906 m^2^ s^−1^	2.660 W m^−1^ K^−1^
Irradiated	2 × 10^15^ cm^−2^	3.263 m^2^ s^−1^	2.982 W m^−1^ K^−1^

### 3.5 Electrical property

**Fig. 5:**
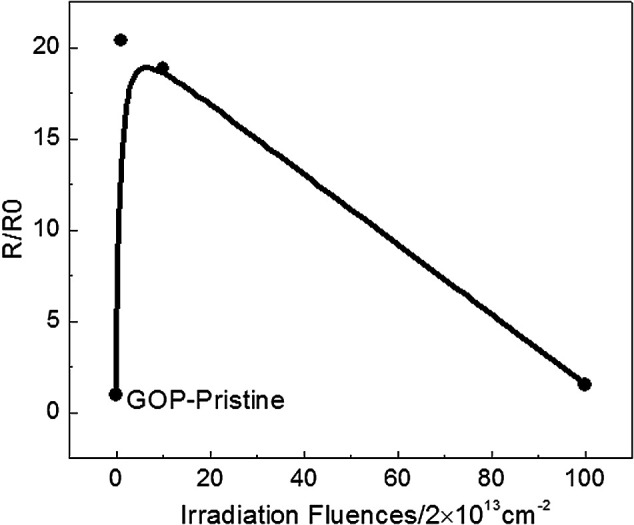
The resistance result of pristine and irradiated GOP.

Now the study of electrical properties for graphene is emphasized on reinforcing the conductivity of polymers, and there are limited studies on pure bulk materials, particularly GO. Here, we have tested the electrical resistance of GOP before and after proton irradiation, as show in Fig. 5. The square resistance of the pristine GOP is 4.08 MΩ and 83.18, 76.92 and 6.28 MΩ after 2 × 10^13^, 2 × 10^14^ and 2 × 10^15^ cm^−2^, respectively. The resistance of GOP materials show remarkable increase and then decrease, while the value also exceeds that of pristine 53.9% after irradiation fluences are made to the highest, similar to thermal conductivity. It is demonstrated that the proton irradiation effect caused the changes in electrical conductivity. The increment in the square resistance after irradiation can be explained by the number of defects. A point defect is the primary effect on electrical conduction degradation in graphene, and the defects increase as well as cause scattering of the charge carriers.^7,10,19,49–52^ The resistance increase in the initial stage may be due to the high defect density, and the decrease may be related to the removal of oxygen-containing functional groups.

### 3.6 Discussion

For the above analysis, the proton irradiation effect on GOP shows the change of structure and property. The XRD results represent a small decrease of *d*-spacing for irradiated GOP with highest fluences 2 × 10^15^ cm^−2^, which is possibly put down to the removal of oxygen-containing functional groups in a significant reduction eventually.^11,53–55^ The presence of a more disordered structure imputes the damage after high fluence proton irradiation. The surface compositions of the GOP show the reduction action event under proton irradiation. It can be explained by the oxygen-containing functional groups decrease for the highest fluence irradiated samples, while the sp^2^ hybridized carbon increase remarkably. The hydroxyl, epoxy and carbonyl groups may be more easily turned to sp^2^ carbon because of better electronic transfers under reduction process induced by proton irradiation. The peak area ratio of *I*_D_/*I*_G_ for 2 × 10^13^ cm^−2^ fluence irradiated GOP increases significantly compared to that of pristine, which indicates the defects introduced under energetic proton irradiation. The ion irradiation can induce polymorphic atomic defects in graphene,^56,57^ which generally contain vacancies, adatom–vacancy, and reconstructed.^18,23,24^ The possible models of vacancies involve the formation of single vacancies (SVs) and double vacancies (DVs), and the adatom–vacancy pair models are constituted by vacancy and adatom, and reconstructions are by Stone–Wales (SW) that take place due to rotating or breaking and the recombination of the C=C bonds.^18^ The energetic ion irradiation damage effect caused on graphene is due to the displacement and excitation of atoms.^29,31,33,34,56,57^ In this experiment, when the 500 keV proton hit against the surface of GOP, the energy of the colliding particles could transform to the target lattice carbon atoms, which caused the carbon atoms to deviate from the initial position, and the defects are formed when these atoms are knocked away. Under the lower 2 × 10^13^ cm^−2^ irradiation fluences, the defects may originate from the vacancy modal in GOP. In the higher fluence irradiated samples, the XPS results indicate the creation of a sp^2^ carbon, which originate from the strong removal of the oxygen-containing functional group through the bond rupture. It can be conjectural that the defects came from the non-six carbon ring. Therefore, it can be concluded that the proton irradiation effect on GOP are defects formation, reduction and structure modification.

In this study, the thermal property of the pristine GOP shows a lower thermal conductivity, which may be caused by the low content of sp^2^ carbon and lots of defects. The heat transport in all bulk carbon allotropes is mostly carried by acoustic phonons, which are attributed to the strong covalent sp^2^ bonding, leading to efficient heat transfer with lattice vibrations.^44–48^ Moreover, the impurities and defects are the powerful primary factors that affect the phonon scatterings in graphene.^49^ We consider that the small thermal conductivity in GOP before and after lower fluence irradiation is attributed to defects and lesser amounts of sp^2^ carbons, respectively. When the irradiation fluences up to 2 × 10^15^ cm^−2^ are used, the slight increase in the thermal conductivity may be induced by two factors. First, the irradiation introduces a higher sp^2^ carbon content, which can improve the efficiency of scattering for phonon transport. Second, the thermal conductivity slightly increases may be due to the removal of large number of oxygen-containing functional groups. The study suggests that a well interface can reduce the interfacial thermal, and good orientation can provide energetic pathways for phonon conduction.^42^ From this point, the removal of oxygen-containing functional groups may be the key factor to improve the quality of interface. The ion irradiation have limited the depth of penetration in graphene,^25,29,31^ and we have calculated the stopped range of 500 keV protons in GOP to be about 8.29 μm, so the change in defects and chemical composition may not obviously be attributed to the increase in depth, which can give a reasonable explanation for thermal properties before and after irradiation GOP. Furthermore, the GOP irradiated with 2 × 10^13^ cm^−2^ exhibits the highest electrical resistance, which is correlated with plenty of oxygen functional groups remaining in the material and the increase in defects. The oxygen-containing functional groups are strong scattering centers, which can affect the electrical transport in graphene, and the defect density also can impact the scattering of charge carriers.^10,50–52^ When the irradiation fluences are increased to the highest 2 × 10^15^ cm^−2^, its more complete reduction action led to the increase in content of sp^2^ and sp^3^ carbon, still retaining many defects, so the resistance is higher than that of pristine GOP but lower than other irradiated ones.

## 4. Conclusion

We have demonstrated the 500 keV proton irradiation effect on GOP produced by Hummers' method. The irradiation brought about the reduction process and the defects. The analysis of XRD indicates the small *d*-spacing caused by relatively complete reduction in GOP after the highest fluences of 2 × 10^15^ cm^−2^. The chemical compositions on the surface of the materials show a decrease in the oxygen-containing functional group and remarkable increase in the sp^2^ carbon, which further confirms the induced reduction by proton irradiation under higher fluence irradiation. The increase in the *I*_D_/*I*_G_ value indicates that a large number of defects are formed under lower 2 × 10^13^ cm^−2^ irradiation fluences, which may be caused by the carbon atoms displacement under collisions of proton. In summary, the defect formation and the reduction action are the main effects in proton effect process. The irradiation effect on thermal conductivity and electrical properties is related to the number of defects, the sp^2^ carbon and oxygen-containing functional group content. The study illustrate that these bulk GO materials can be used as thermal or electrical conductivity coating, or electronic devices in future space missions.

## Conflicts of interest

There are no conflicts to declare.

## Supplementary Material

RA-009-C9RA05389A-s001
