# Supplementary material for: The effect of proton irradiation on the properties of a graphene oxide paper
Source: RSC Adv. 2019 Sep 26;9(52):30519–25. doi: 10.1039/c9ra05389a (PMC9072190; doi:10.1039/c9ra05389a)

Electronic Supplementary Material (ESI) for RSC Advances.
This journal is © The Royal Society of Chemistry 2019

Supplementary information

1. The cross section SEM image of GOP

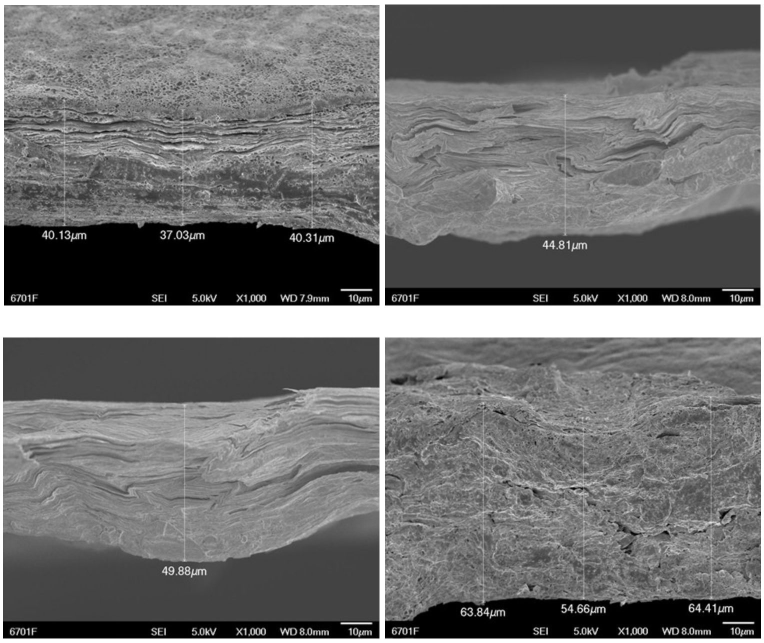

2. The size image of GOP

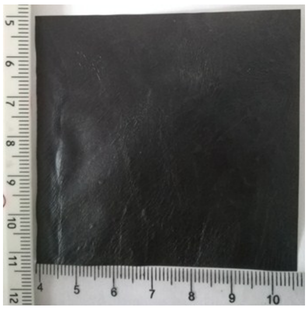

3. The 500 keV protons distribution dependent on target GOP depth was calculated by SRIM

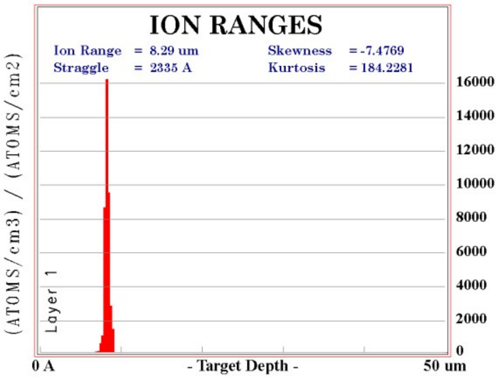

Supplement: RA-009-C9RA05389A-s001 [file RA-009-C9RA05389A-s001.pdf]
